# Combination Therapy with Losartan and α-Tocopherol in Acute Ureteral Obstruction-Induced Renal Excretory Dysfunction and Acidification Defect

**Published:** 2014-07

**Authors:** Izadpanah Gheitasi, Seyed Mostafa Moosavi

**Affiliations:** Department of Physiology, School of Medicine, Shiraz University of Medical Sciences, Shiraz, Iran

**Keywords:** Alfa-tocopherol, Losartan, Renal tubular acidosis, Ureteral obstruction

## Abstract

**Background:** Previous study by the authors showed that a-tocopherol prevents oxidative stress but would not improve depressed excretory variables in post-obstructed kidney (POK) after release of 24-h unilateral ureteral obstruction (UUO). This study is a supplementary investigation on the effects of a-tocopherol combined with an antagonist of angiotensin-II type-1 (AT_1_) receptor on renal dysfunction following release of acute UUO.

**Methods: **The left ureter was ligated in different groups of male Sprague-Dawley rats that received normal saline, losartan or losartan/a-tocopherol (n=6 in each group). After releasing 24-h UUO, urine of each kidney was separately collected under paraffin during 1-3 h of post-release period and then both kidneys were removed for measuring malondialdehyde (MDA) and ferric reducing/antioxidant power (FRAP).

**Results:** Losartan**-**treatment decreased MDA and increased FRAP, creatinine-clearance and sodium-reabsorption in POK, while co-treatment with losartan and a-tocopherol not only augmented improvement in these variables but also elevated potassium-excretion, free-water reabsorption and urine-osmolality. However, UUO-induced fall in urinary pCO_2_ and rise in pH and bicarbonate-excretion of POK were ameliorated equally with losartan and losartan/a-tocopherol.

**Conclusion:** Activation of AT_1_-receptor contributes to the development of renal distal acidification defect induced by acute ureteral obstruction. The co-treatment with losartan and a-tocopherol showed that their effects on preventing oxidative stress along with ameliorating glomerular filtration and tubular fluid-delivery in POK could lead to improvement in tubular transport of sodium and potassium as well as urine-concentrating ability at the early post-release period.

## Introduction

Unilateral ureteral obstruction (UUO) even with a short duration of 24-h leads to a severe decline in renal blood flow (RBF), glomerular filtration rate (GFR), solutes excretion, urine-concentrating ability and urinary acidification during early hours following the release of blockade in the post-obstructed kidney (POK) while producing minimal histological changes.^1^^-^^3^ However, the contralateral non-obstructed kidney (NOK) can perform compensatory hyper-function to prevent solutes retention, volume expansion, acidemia and uremia during obstruction and post-release periods.^1^^,^^4^ It is well-known that acute ureteral obstruction disturbs tubular transport of solutes and water through suppressing membrane expression of several major Na^+^-carriers and Na^+^/K^+^-ATPase pump as well as aquaporin (AQP) water channels and urea transporters along the different segments of tubules.^4^^-^^7^ In addition, collecting duct has been documented to be the main site of ureteral obstruction-induced impairment in acid excretion, in which activity of H^+^-secretory pumps is inhibited without change in their abundance.^8^^-^^11^

In the collecting duct, a-intercalated cells reabsorb HCO_3_^-^ via H^+^-secretion by H^+^-ATPase and H^+^/K^+^-ATPase. In normal conditions, HCO_3_^-^ react with secreted H^+^ very quickly in the lumen to generate H_2_CO_3_ that its slow dehydration to form CO_2_ in the absence of luminal membrane carbonic anhydrase causes pH disequilibrium.^12^ Thus, CO_2_ will be formed in areas of collecting system and urinary tract (where surface-volume relationship is unfavorable for its back diffusion) resulting in the elevation of urinary pCO_2_.^9^^,^^12^ In several in vivo studies, the fall of urinary pCO_2_ in POK was regarded as an index of suppressed capacity of distal H^+^-pumps to secrete H^+^ that was associated with rise in urinary pH and bicarbonate excretion.^9^^,^^10^^,^^11^

In recent studies on rats subjected to 24-h UUO followed by 2 h post-release period, it was observed that a-tocopherol as the most potent lipid-soluble antioxidant could prevent oxidative stress in the POK. However, it did not have any improving effect on its disturbed glomerular filtration, solutes excretion and urine-concentrating ability^1^ as well as distal acidification defect.^13^ Therefore, it was suggested that oxidative stress probably might not contribute in the early development of renal haemodynamic and excretory dysfunctions induced by ureteral obstruction. On the other hand, there could be another possibility that the maintenance of reduced RBF and GFR (i.e. low delivery of solutes and water into the tubules) preventing the improved renal redox state to be able to ameliorate ureteral obstruction-induced decrement of tubular transport. To test this hypothesis, losartan as a selective antagonist of angiotensin-II (Ang-II) type-1 (AT_1_) receptor is used in this study since it is well-known that Ang-II,^14^^-^^16^ through its AT_1_-receptor increases renal arteriolar constriction to reduce RBF and GFR in ureteral obstruction.^7^^,^^17^ Consequently, the present study was designed to investigate the effects of losartan alone and combined with a-tocopherol on changes in haemodynamics, solutes excretion, urine-concentrating ability, acid-base excretion and oxidative balance at the early hours following release of 24-h UUO in both POK and NOK.

## Materials and Methods

*Experimental Animals*

This experimental interventional study was done on 24 male Sprague-Dawley rats weighing 280-320 g (provided by the Centre of Experimental Animals, Shiraz University of Medical Sciences, Shiraz, Iran). Few days prior to the surgery, the rats were kept in cages with free access to water and standard rodent diet at a temperature-controlled room (23±1°C) with 12 h artificial light and dark cycle in the Department of Physiology. All procedures were approved by the institutional ethics committee of Shiraz University of Medical Sciences, which follows the guidelines for the care and handling of animals prepared by the Iranian Ministry of Health and Medical Education and in accordance with the international conventions on animal experimentation.  

*Induction of Unilateral Ureteral Obstruction and Experimental Groups*

Each rat was anaesthetized by diethyether and the left ureter was ligated by 4-0 silk suture at two points under surgical microscope (Olympus Corporation, Japan). The animal was then allowed to recover from the anaesthesia prior to returning to its dedicated cage.^18^

The rats were divided into four groups (n=6 in each group). There were 1-mL intraperitoneal (i.p.) injections of either normal saline in the control group or normal saline containing losartan potassium (20 mg/kg of body weight; donated by Razak Laboratory Co., Iran) in the Los group at 36 h and 12 h before and 12 h after UUO-induction.^19^^-^^21^ This dose of losartan was hypotensive (table 1) but MAP was in the autoregulatory range of rats^19^^,^^22^ while lower doses with different durations that were examined in the preliminary experiments could not improve GFR of POK. Combination therapy with losartan and a-tocopherol was done in the Los+AT group.  The losartan was administered in the same manner as the Los group while there were also 1-mL i.p. injections of olive oil containing a-tocopherol acetate (50 mg/kg of body weight; Sigma, UK) 6 h before and 9 h after UUO-induction.^1^^,^^18^ In the sham group, the left ureter was exposed and manipulated but not occluded while 1-ml normal saline was i.p. injected 36 h and 12 h before and 12 h after sham-surgery.

**Table 1:** Effects of losartan alone and combined with a-tocopherol on plasma variables and arterial pressure following release of acute unilateral ureteral obstruction

** Variables ** **Groups**	** Plasma Na^+^** **(mmol/L)**	** Plasma K^+^** **(mmol/L)**	** Plasma creatinine ** **(mg/dL)**	** Plasma urea nitrogen ** **(mg/dL)**	** Plasma osmolality ** ** (mosm/kgH_2_O) **	** Mean arterial pressure ** **(mmHg)**
Sham	147.8±0.3	3.60±0.06	0.59±0.03	18.3±0.9	298.2±1.8	109.7±5.5
Control	148.3±0.3	3.80±0.11	0.77±0.03**^†^**	24.2±1.0	302.7±1.8	117.7±3.2
Los	147.1±0.7	3.68±0.03	0.68±0.03****	26.3±2.7****	300.8±2.0	99.2±1.9**^ † §§ ^**
Los+AT	147.2±0.4	3.83±0.06	0.70±0.03****	25.9±3.0	298.8±1.5	96.6±3.1**^† §§§^**

Data (means±SEM) are the average of two measured values at the beginning and the end of clearance period for each plasma variable as well as average of mean arterial pressure during the clearance period of 1-3 h after release of 24-h UUO in the different groups of rats. †P<0.05, *vs*. the sham group. ANOVA followed by Duncan and then LSD. §§P<0.01, §§§P<0.001, the treated-groups *vs*. the control group

*Experimental Protocol*

After 23 h elapse of UUO-induction, each rat was re-anaesthetized by pentobarbital sodium (60 mg/kg of body weight, i.p.; Sigma). Tracheotomy tube was inserted and After tracheotomy, a mask connected to oxygen tank was placed on it to maintain arterial O_2_ pressure higher than 80 mmHg.  Then the probe of thermistor was put into the rectum to keep its temperature at 37±1°C. A cannula was inserted into the right femoral vein to infuse normal saline at 3 mL/h throughout the experiment by a syringe-infusion pump, as well as bolus doses of pentobarbital as necessary. The right femoral artery was also cannulated and connected to a pressure transducer (MLT844; ADInstruments, Australia) for continuous recording (PowerLab/4SP data acquisition system, ADInstruments, Australia) of arterial pressure. Afterward, the right ureter was cannulated and a cannula was inserted into the left ureter in between the two ligatures. The left ureteral obstruction was released at exactly 24-h after UUO-induction and the animal was then allowed to have 1 h of equilibration.^1^

A clearance period with 2 h duration at 1-3 h following the release of UUO was taken to collect urine from each kidney in a separate pre-weighed container under 30 mm paraffin. Two arterial blood samples (1-mL) were taken at the beginning and at the end of clearance period where 0.2 mL of each sample was quickly analyzed for its acid-base and gases. The remainder of each sample was centrifuged and the plasma was stored at -20^o^C until assayed. The collected urine of each kidney beneath paraffin was brought out and its CO_2_ pressure and acidity were immediately analyzed. The remainder of urine was diluted and kept in the refrigerator. Finally, both kidneys were removed, frozen in liquid nitrogen and preserved at -80°C for later evaluation of their redox indices. Rats were sacrificed by injecting an overdose of pentobarbital anaesthetic.

*Analytical Methods*

Arterial pH (pH_a_), O_2_ tension (p_a_O_2_), CO_2_ tension (p_a_CO_2_) and bicarbonate concentration ([HCO_3_^-^]_a_) as well as urinary CO_2_ tension (p_u_CO_2_) were determined by an Easy blood-gas analyzer (Medica Corporation, USA). The pH of urine (pH_u_) was measured by argus-X pH-meter (Sentron, Netherlands). The urinary HCO_3_^-^ concentration was calculated by using Henderson-Hasselbalch equation and taking solubility coefficient of CO_2_ for urine (0.0309). pK of urinary bicarbonate buffer was obtained according to the formula of 6.33–0.5([Na^+^]_u_+[K^+^]_u_)^1/2^, taken urine concentrations of Na^+^ and K^+^ in mol/L.^23^

Plasma and urine samples were assayed for concentrations of creatinine and urea nitrogen by an autoanalyzer (Technicon Instruments, USA), Na^+^ and K^+^ with an EasyLyte analyzer (Medica Corporation, USA) and osmolality by using a cryoscopic osmometer (Gonotec GmbH, Germany). The volume of collected urine from each kidney during the clearance period was measured gravimetrically and its urine flow rate per gram of kidney weight (V^0^; mL/min.gkw) was calculated. Creatinine clearance (C_Cr_) as an estimate of GFR, absolute excretion of Na^+^ (U_Na_V^0^), K^+^ (U_K_V^0^), urea (U_urea_V^0^) and HCO_3_^-^ (U_HCO3_V^0^), fractional excretion of Na^+^ (FE_Na_), K^+^ (FE_K_), urea (FE_urea_) and HCO_3_^-^ (FE_HCO3_) as well as osmolar clearance (C_osm_) and free-water reabsorption (T^c^_H2O_) were calculated by standard formulae. Additionally, the ratios of C_osm_ to C_Cr_ (C_osm_/C_Cr_), T^c^_H2O_ to C_Cr_ (T^c^_H2O_/C_Cr_) and T^c^_H2O_ to C_osm_ (T^c^_H2O_/C_osm_) were determined.

*Ferric Reducing/Antioxidant Power (FRAP) and Malondialdehyde (MDA) Assays*

To evaluate renal redox state, each frozen kidney sample was quickly weighed and homogenized in ice-cold phosphate-buffered saline (1:10 W/V). Then, as it was previously described in details,^18^ the renal tissue levels of malondialdehyde (MDA) in nmol/gkw (the final product of lipid peroxidation and FRAP in mmol/gkw as a direct measure of total antioxidant activities of all defence mechanisms) were determined by using a spectrophotometer (Spectrolab, England).

*Statistical Analysis*

Data are expressed as means±SEM and comparisons between the right and left renal variables in each group were assessed using the student’s paired *t *test. The blood parameters and variables of equivalent kidneys were compared between groups by one-way ANOVA followed by Duncan’s post-hoc test. Then, the least significant difference (LSD) test was performed for determining the exact level of P values. SPSS 11.5 software was used for all data analyses and significance was taken at P<0.05.

## Results

*Plasma Variables and Mean Arterial Pressure*

As shown in table 1, the average of each plasma variable and mean arterial pressure (MAP) during whole 1-3 h after release of 24-h UUO in the control group were not statistically different from those of the sham group at the equivalent period, except for the higher concentration of plasma creatinine. Treatment with losartan alone or in combination with a-tocopherol prevented the rise of plasma creatinine while MAP in the Los and Los+AT groups significantly decreased with respect to the control and sham groups.

*Renal Redox Status, Glomerular Filtration and Urine Flow*

**Figure 1:**
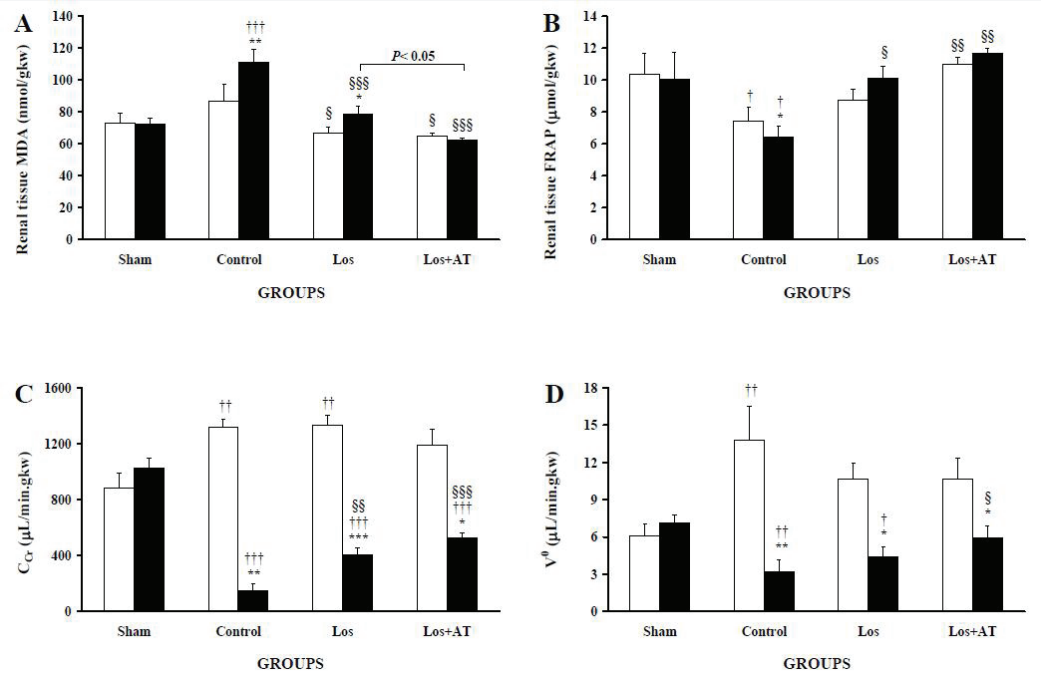
The levels of A) malondialdehyde (MDA) and B) ferric reducing/antioxidant power (FRAP) at the end, and also C) creatinine clearance (C_Cr_) and D) urine flow rate (V0) are expressed as means±SEM for the right non-obstructed kidney (□) and the left post-obstructed kidney (■) during 1-3 h after release of 24-h UUO in the different groups of rats. *P<0.05, **P<0.01, ***P<0.001, for comparison between two kidneys in each group. †P<0.05, ††P<0.01, †††P<0.001, *vs*. equivalent kidney of the sham group. §P<0.05, §§P<0.01, §§§P<0.001, equivalent kidney of the treated-groups *vs.* the control group.

Losartan and losartan/a-tocopherol decreased MDA (figure 1A) in POK and NOK. Also FRAP (figure 1B) was increased in POK of the Los group and in POK and NOK of the Los+AT group compared with those of the control group, so that MDA and FRAP in both kidneys of each treated-group reached to their levels in the equivalent kidneys of sham group. However, POK of the Los group had higher MDA than the Los+AT group as well as its contralateral NOK.

Compared with the control group, C_Cr_ (figure 1C) of POK was elevated by 2.8-fold and 3.7-fold in the Los and Los+AT groups respectively. However, C_Cr_ in POK of both treated-groups were still lower than the left kidney (LK) of sham group as well as their own NOK. In addition, C_Cr_ of NOK in the Los group remained higher than the right kidney (RK) of sham group but not in the Los+AT group. As shown in figure 1D, V^0 ^of POK was raised in the Los+AT with respect to the control group and became equal to its level in the LK of sham group, however it was not changed in the Los group. Moreover, V^0^ in NOK of the Los and Los+AT groups were not significantly (both P=0.078) higher than that of the RK of sham group while V^0^ was lower in the POK than the NOK in each of the obstructive groups.

*Sodium, Potassium and Urea Excretion*

**Figure 2:**
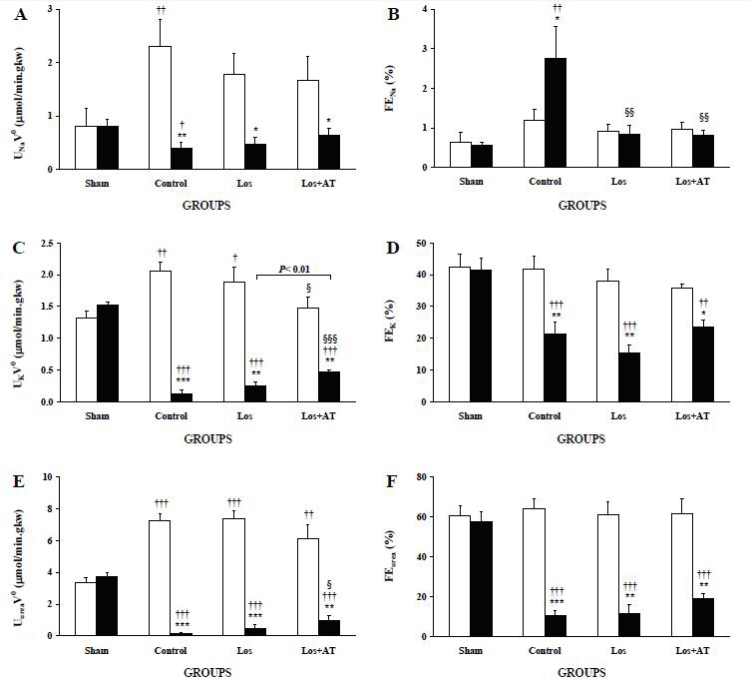
The levels of A) absolute sodium excretion (U_Na_V0), B) fractional sodium excretion (FE_Na_), C) absolute potassium excretion (U_K_V0), D) fractional potassium excretion (FE_K_), E) absolute urea excretion (U_urea_V0), and F) fractional urea excretion (FE_urea_) are expressed as means±SEM for the right non-obstructed kidney (□) and the left post-obstructed kidney (■) during 1-3 h after release of 24-h UUO in the different groups of rats. *P<0.05, **P<0.01, ***P<0.001, for comparison between two kidneys in each group. †P<0.05, ††P<0.01, †††P<0.001, *vs*. equivalent kidney of the sham group. §P<0.05, §§P<0.01, §§§P<0.001, equivalent kidney of the treated-groups *vs*. the control group.

Losartan prevented U_Na_V^0^ (figure 2A) to be statistically reduced in POK and elevated in NOK (P=0.064 and 0.096 respectively) from the equivalent kidneys of sham group. It also decreased FE_Na_ (figure 2B) in POK from that of the control group to reach to its level in the LK of sham group. Nonetheless, it did not affect U_K_V^0^ (figure 2C), FE_K_ (figure 2D), U_urea_V^0^ (figure 2E) and FE_urea_ (figure 2F) in both kidneys. However, losartan/a-tocopherol resulted in the reduction of FE_Na_ in POK and U_K_V^0^ in NOK and likewise the elevation of U_K_V^0^ and U_urea_V^0^ in POK with respect to those of the control group. It also prevented the rise in U_Na_V^0^ of NOK from the RK of sham group plus having higher U_K_V^0^ in POK than that of the Los group.

*Osmolar Clearance and Urine-Concentrating Ability*

**Figure 3:**
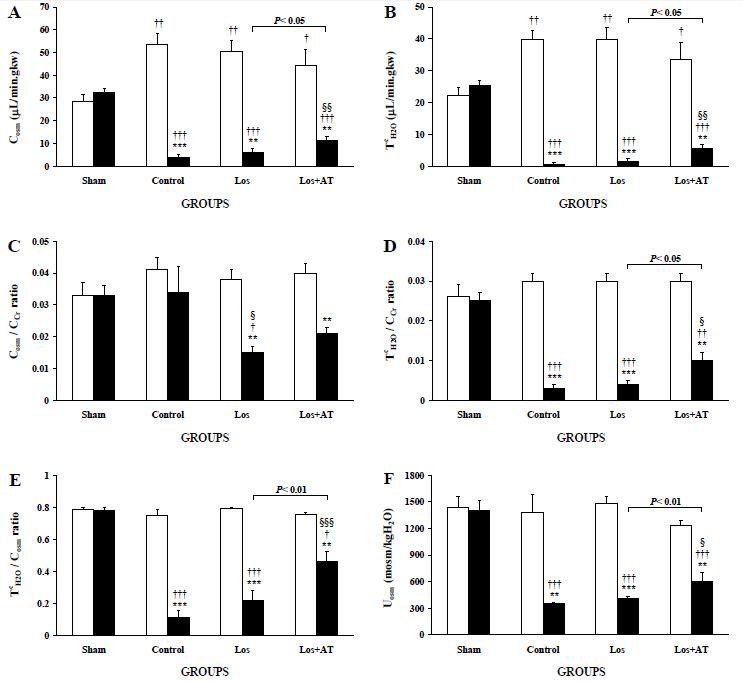
The levels of A) osmolar clearance (C_osm_), B) free-water reabsorption (Tc_H2O_), C) C_osm_ to C_Cr_ ratio (C_osm_/C_Cr_), D) Tc_H2O_ to C_Cr_ ratio (Tc_H2O_/C_Cr_), E) Tc_H2O_ to C_osm_ ratio (Tc_H2O_/C_osm_), and F) urinary osmolality (U_osm_) are expressed as means±SEM for the right non-obstructed kidney (□) and the left post-obstructed kidney (■) during 1-3 h after release of 24-h UUO in the different groups of rats. *P<0.05, **P<0.01, ***P<0.001, for comparison between two kidneys in each group. †P<0.05, ††P<0.01, †††P<0.001, *vs*. equivalent kidney of the sham group. §P<0.05, §§P<0.01, §§§P<0.001, equivalent kidney of the treated-groups *vs*. the control group.

Losartan-treatment could not change C_osm_ (figure 3A) and T^c^_H2O_ (figure 3B) in POK whereas they were increased in the Los+AT group compared with the control and Los groups. The compensatory increases of C_osm_ and T^c^_H2O_ in NOK of the control group with respect to the RK of sham group remained unchanged in both treated-groups. Hence C_osm_ and T^c^_H2O_ were lower in the POK than the NOK in all obstructive groups.

C_osm_/C_Cr_ (figure 3C) of POK decreased significantly in the Los group and insignificantly in the Los+AT group from those of the control group and the LK of sham group. C_osm_/C_Cr_ of NOK in both Los and Los+AT groups was equal to those of the control group and the RK of sham group but higher than their own POK. On the other hand, losartan did not affect T^c^_H2O_/C_Cr_ (figure 3D), T^c^_H2O_/C_osm_ (figure 3E) and U_osm_ (figure 3F) in both kidneys whereas their levels in POK of the Los+AT group increased from those of the control and Los groups even though they were still lower than those of the LK of sham group and its NOK.

*Arterial Acid-Base Variables*

Table 2 shows that the values for pH_a_, p_a_CO_2_, p_a_O_2_ and [HCO_3_^-^]_a_ during 1-3 h following release of 24-h UUO in the control group and both treated-groups are not different and are equal to those of the sham group at the equivalent period.

**Table 2:** Effects of losartan alone and combined with a-tocopherol on arterial acid-base variables following release of acute unilateral ureteral obstruction

** Variables** ** Groups **	**Arterial pH**	** Arterial CO_2_ (mm Hg) **	** Arterial O_2_ (mm Hg) **	** Arterial HCO_3_^-^ (mmol/L) **
Sham	7.43±0.02	36.6±2.8	86.0±2.8	24.2±0.7
Control	7.44±0.01	37.1±1.9	104.0±12.5	25.1±0.4
Los	7.41±0.02	39.4±0.7	97.3±8.7	25.0±0.6
Los+AT	7.42±0.01	39.9±1.3	93.1±2.4	25.5±0.7

Data (means±SEM) are the average of two measured values for each arterial variable at the beginning and the end of a clearance period during 1-3 h after release of 24-h UUO in the different groups of rats. ANOVA followed by Duncan and then LSD.

*Renal Acid-Base Excretion*

**Figure 4:**
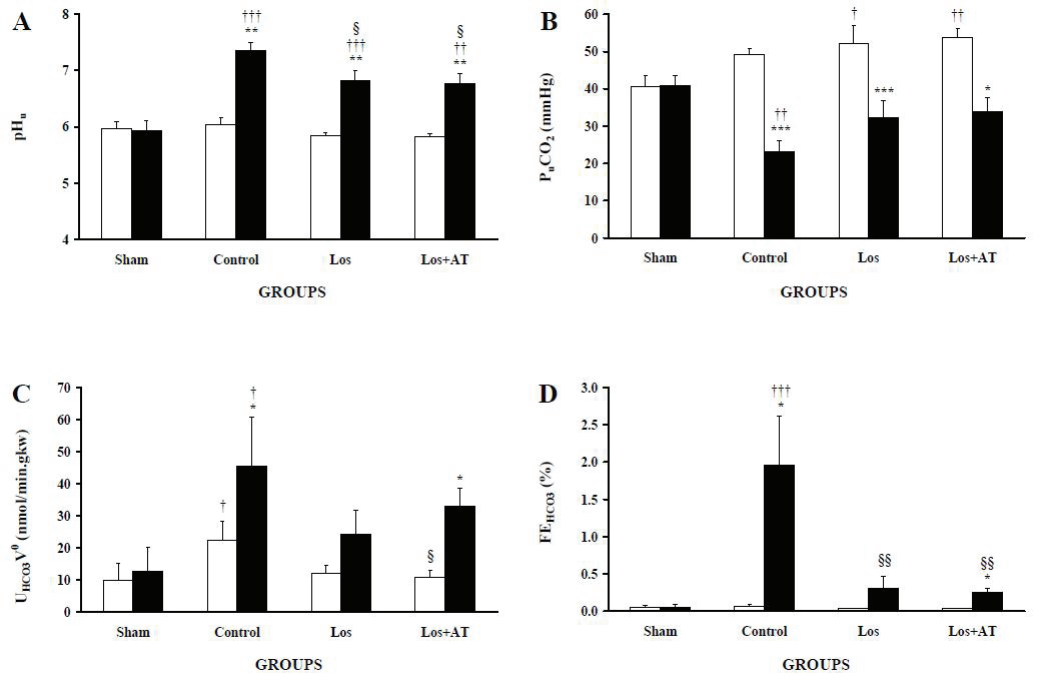
The levels of A) urinary pH (pH_u_), B) urinary carbon dioxide pressure (p_u_CO_2_), C) absolute bicarbonate excretion (U_HCO3_V0), and D) fractional bicarbonate excretion (FE_HCO3_) are expressed as means±SEM for the right non-obstructed kidney (□) and the left post-obstructed kidney (■) during 1-3 h after release of 24-h UUO in different groups of rats. *P<0.05, **P<0.01, ***P<0.001, for comparison between two kidneys in each group. †P<0.05, ††P<0.01, †††P<0.001, *vs*. equivalent kidney of the sham group. §P<0.05, §§P<0.01, §§§P<0.001, equivalent kidney of the treated-groups *vs*. the control group.

Losartan and losartan/a-tocopherol equally affected all renal acid-base excretory variables in both kidneys. The pH_u_ (figure 4A) of POK in the Los and Los+AT groups were reduced from the control group but pH_u_ in their NOK remained unchanged. However, pH_u_ of POK in both treated-groups were still higher than the LK of sham group as well as their own NOK. Although p_u_CO_2_ (figure 4B) in POK of the Los and Los+AT groups were not statistically elevated from the control group, they were not also statistically lower than p_u_CO_2_ of the LK in sham group. This is while NOK in both treated-groups had higher p_u_CO_2_ than the RK of sham group. The reduction of U_HCO3_V^0^ (figure 4C) in POK of the Los and Los+AT groups from the control group were not significant but also were not significantly higher than that of the LK in sham group. Moreover, U_HCO3_V^0^ of NOK was lower in the Los+AT group than the control group as well as its own POK. FE_HCO3_ (figure 4D) of POK in both treated groups entirely declined compared with the control group and reached to its level in the LK of sham group while FE_HCO3_ in their NOK remained unchanged.

## Discussion

The blockage of AT_1_-receptors by losartan blunted the fall of GFR in POK of the Los group which in conjunction with compensatory increase in GFR of NOK, prevented the rise of plasma creatinine. On the other hand, other studies suggested that the direct mechanical stretch, ischaemia and increased Ang-II probably stimulated tubules to produce cytokines which were operant in down-regulating antioxidant enzymes to induce renal oxidative stress in the setting of UUO, especially at the early phase.^24^^,^^25^ In support, losartan prevented development of oxidative stress in the POK of Los group which might also contribute to reduction of its arteriolar constriction. It has been found that superoxide reacts with NO to form peroxynitrite which induces endothelial dysfunction and augments vasoconstriction.^26^ However, administration of a-tocopherol alone that normalized redox state of the POK did not improve its reduced GFR.^1^ Also, losartan/a-tocopherol that caused more reduction in MDA and elevation in FRAP of POK, did not statistically elevate its C_Cr_ to higher value than that of the Los group but with P=0.08. It is necessary to mention that the activated autoregulatory mechanisms by losartan-induced hypotension^22^ might interfere with the responses of glomerular arterioles to the improvement in oxidative state for elevating GFR.

In the Los group, the increased GFR was associated with the reduction of FE_Na_ to normal level in the POK which definitely indicated increment of tubular Na^+^-reabsorption. The fall in MAP, despite of being in the autoregulatory range, probably reduced peritubular capillary pressure to slightly enhance tubular reabsorption.^27^ Additionally, Jensen et al. showed that treatment with an AT_1_-recptor antagonist partially prevented the acute ureteral obstruction-induced downregulation in Na^+^-phosphate cotransporter type-2 (NaPi-2) at the proximal tubule, Na^+^-K^+^-2Cl^-^ cotransporter type-2 (NKCC2) at the thick ascending limb, AQP2 at the collecting duct and attenuated the reduction in Na^+^ and water reabsorption.^7^ However, in spite of enhanced tubular Na^+^-reabsorption, urinary concentrating ability was not improved in POK of the Los group. It can be envisaged that losartan-induced increment in medullary blood flow probably led to washout of the increasingly reabsorbed sodium^28^ and did not allow solutes to be accumulated in medullary interstitium of POK. This is supported by no change in its indirect marker of U_osm_, for driving water-reabsorption, and hence T^c^_H2O_, T^c^_H2O_/C_Cr_ and T^c^_H2O_/C_osm_ were not increased. Moreover, losartan-treatment did not also affect excretion of K^+^ and urea in the POK and the fall in its C_osm_/C_Cr_ was due to enhanced Na^+^-reabsorption. In contrast, losartan/a-tocopherol not only increased tubular Na^+^-reabsorption but also elevated U_K_V^0^, U_urea_V^0^ and C_osm_ in the POK. Consequently, compensatory excretion of solutes in the NOK was somehow mitigated. It is important to note that, although the increase in GFR and filter-load had a main role in enhanced absolute excretion of solutes in POK of the Los+AT group, its levels of FE_K_, FE_urea_ and C_osm_/C_Cr_ indicated that tubular secretions of K^+^ and urea were more likely ameliorated by losartan/a-tocopherol compared with losartan alone. Interestingly, urinary concentrating ability was partially improved in POK of the Los+AT group and increments in its T^c^_H2O_/C_Cr_ and T^c^_H2O_/C_osm_ implied that the elevation in T^c^_H2O_ was not due to only raised GFR and solute delivery to distal nephron. There were definitely improvements of the counter current multiplication mechanism in the Henle’s loop and mechanism of solute-free water reabsorption in the collecting duct. Hence, losartan/a-tocopherol could potentiate accumulation of Na^+ ^and urea in medullary interstitium of the POK to establish its hyperosmolarity (as evidenced by rise in U_osm_) which resulted in the elevation of T^c^_H2O_. On the other hand, in spite of elevated T^c^_H2O_, the V^0^ in POK of the Los+AT group increased to the level of sham group which was due to rise in C_osm_, since V^0^=C_osm_-T^c^_H2O_. In general, comparison between the results obtained from the Los+AT and Los groups indicates that oxidative stress definitely plays a role in down-regulating tubular transport mechanisms during acute ureteral obstruction and its effect is augmented with AT_1_-receptor activation. Moreover, losartan with its improving effect on GFR and hence making sufficient availability of solutes and water in the tubules of POK, resulted that a-tocopherol with normalizing renal oxidative state was able to potentiate amelioration of suppressed Na^+^-reabsorption, tubular transport of K^+^ and urea and urinary concentrating ability following the release of 24-h UUO in the Los+AT group.

The fall of p_u_CO_2_ in POK of the control group was an index of suppressed capacity of distal H^+^-pumps to secrete H^+^ that led to rise in its pH_u_, U_HCO3_V^0^ and FE_HCO3_. This is also observed by other researchers.^8^^,^^9^^,^^12^ Several studies showed that acute ureteral obstruction suppressed the activity of distal nephron H^+^-secretory pumps but not their expression. This was more likely exerted by local inhibitory factors that were up-regulated in the obstructed kidney.^8^^-^^11^ In an in vitro experiment on renal medulla homogenates and microdissected inner medullary collecting duct segments obtained from rats subjected to 24-h UUO, it was found that Ang-II through its AT_1_-receptor stimulated inducible nitric oxide synthase (iNOS) activity to increase NO levels in renal medulla, which was involved in the inhibition of H^+^-ATPase activity at inner medullary collecting duct segments.^29^ Importantly, the elevations of pH_u_, U_HCO3_V^0^ and FE_HCO3_ as well as the fall of p_u_CO_2_ were mitigated in the POK of Los group. These are new findings for the in vivo condition and in accordance with results of the only previous in vitro study^29^ to confirm that endogenous activation of AT_1_-receptor plays a role in the suppression of H^+^-secretory pumps to develop defected renal distal acidification in acute ureteral obstruction. Interestingly, NOK in both Los and Los+AT groups showed higher p_u_CO_2_ and equal U_HCO3_V^0^ with respect to the RK of sham group. This indicates that AT_1_-receptor even in NOK had a bit of inhibitory effect on distal H^+^-secretory pumps, which might be through iNOS activation, as its expression was shown to be raised during 24-h UUO in both kidneys.^30^ On the other hand, the intensities of improvements in all acid-base excretory variables of POK in the Los+AT group were in exact similarity to those of the Los group. Also, it was previously observed that treatment with a-tocopherol alone did not affect any acid-base variables in both kidneys after release of 24-h UUO.^13^ Therefore, it can be concluded that oxidative stress most likely does not contribute to acute ureteral obstruction-induced impairment in renal distal acidification.

## Conclusion

Endogenously activated AT_1_-recpotor plays a major role in inhibiting H^+^-secretion at the distal nephron to suppress acid excretion during early hours following release of 24-h UUO. The combined therapy with losartan and a-tocopherol demonstrated that oxidative stress contributes in the early development of acute ureteral obstruction-induced disturbances in Na^+^-reabsorption, tubular handling of K^+^ and urea, and urinary concentrating ability but not distal acidification defect.
